# Nursing care from the perspective of subcultural identities: a reflective essay

**DOI:** 10.1590/1980-220X-REEUSP-2025-0140en

**Published:** 2025-10-20

**Authors:** Lilian Cristiane Gomes, Maria Lúcia do Carmo Cruz Robazzi, Sérgio Valverde Marques dos Santos, Fábio de Souza Terra, Mônica La Salette da Costa Godinho, Cristiane Aparecida Silveira, Ana Kelley de Rezende, João Vitor Andrade, Namie Okino Sawada

**Affiliations:** 1 Universidade Federal de Alfenas, Escola de Enfermagem, Alfenas, MG, Brazil.

**Keywords:** Social Identification, Cultural Diversity, Stereotyping, Nursing Care, Cultural Competency

## Abstract

This theoretical-reflective study investigated Identity Diversity and Nursing Care, based on materials selected from PubMed/MedLine and FurScience, providing a critical reflection on the topic. Two categories were developed: “New Identities: Their Expressions and Social Implications,” which addresses the construction of social and subcultural identities in groups with common interests and how these identities influence society, and “Nursing Care from the Perspective of New Identities,” which highlights the importance of knowledge about subcultures and social identities in care, especially for adolescents and young people. This understanding favors humanization, strengthens the therapeutic bond, and minimizes barriers in nursing care. The new identities are seen as dynamic sociocultural constructions, whose theoretical reflection highlights the importance of demystifying stigmas and promoting humanized care. In addition, it emphasizes nursing cultural and ethical competencies in redefining care for stigmatized people, encouraging debates in professional health training.

## INTRODUCTION

In a globalized world, permeated by increasingly rapid and significant innovations and technological advances^(1)^, new forms of socialization and social aggregation emerge, which, in turn, produce their own cultures or subcultures^(2–3)^. The term “subculture” refers to the values, beliefs, attitudes, identity, and lifestyles of a group within society^(4)^. As an example, there are the nerd and geek subcultures, in which the followers are people exceptionally involved or interested, respectively, in technologies/exact sciences and pop culture/invented worlds (comics, anime and manga, cosplay, among others), being specialists in these areas. The geek subculture, in particular, has gained great popularity and in it, each area of interest constitutes a “fandom”, that is, a community of fans or supporters^(5,6,7,8)^.

Before the digital age, those identifying themselves as “geeks” seemed isolated, both in their own interests and from their peers. This is because many geek activities (such as reading, writing, and playing games) were considered inherently “less social” because they were mostly done alone. Even the “geeks” who lived in big cities faced difficulties in finding and connecting with others who participated in the same fandoms/communities^(7)^. The attachment to and performance of these activities were seen, in some ways, as antisocial behavior or as a sign of indifference to social interactions, resulting in negative stereotypes^(5,7,8)^. The advent of the Internet, however, has given people the opportunity to connect freely, without restrictions of time, distance, gender, or age. Just like in real life, people with common interests began to gather in virtual spaces to socialize. In general, teenagers and young people adapted more quickly to the new scenario, helped by their ability to assimilate technology with minimal effort. This opened up a range of possibilities for young people to choose from when defining and presenting themselves to the world^(7)^.

When reflecting on the fact that interests and tastes are changeable, it becomes understandable that activities previously tainted by too much social stigma can now gain social capital online^(7)^. This fact is so genuine that, since the last decade, geek activities have become blockbusters, multi-billion dollar industries, and a far-reaching subculture, with their own brands, fashion trends, and celebrities. It is also necessary to reflect that, despite being commonly associated with recreation or entertainment, the geek subculture constitutes an essential component of the lives of many enthusiasts as it influences their emotional and behavioral experiences and even their perceptions about health and self-care^(5)^.

It is in this subcultural context that the adoption of new identities, called “unconventional”, “atypical”, or “non-human”, emerges extensively^(9)^. Although the phenomenon of globalization and the profusion of digital technologies have fostered a greater movement of ideas, as well as a greater circulation of people and information^(1)^, little is still known about this “neo-identity” universe. It is important to emphasize that the term “new identities” used here does not refer to its chronology/temporal novelty, but to the scarce discussion it raises in the scientific literature in the health area, which contributes to the persistence of prejudice, stereotypes, and misinformation. This situation, besides raising relevant questions, requires the attention of health professionals, especially nursing professionals, as a profession focused on the comprehensive care of human beings and, often, responsible for the initial reception in health services. Considering the specificity of their functions, it is essential that these professionals are aware of these cultural singularities to recognize, understand, and respect practices and identities of digital and media subcultures, such as the geek one, to establish therapeutic bonds supported by an empathetic and understanding perspective with those receiving their care.

Thus, this study aims not only to clarify the definitions underlying the new identities and their social implications, but, mainly, to provide a theoretical-reflective framework that can assist nurses and nursing teams in approaching and providing care to people, as well as in supporting and clarifying families, so as to prevent and/or minimize the bias of stigmatization and possible forms of psycho-emotional suffering. Thus, the objective of the present study is to discuss the new identities, the reasons that support them, and their repercussions for nursing care.

## METHOD

This is a theoretical-reflective essay, prepared through a literature review, in a narrative form and based on the following concerns: what are the new identities and what influences and/or motivates them? What reflections emerge about nursing care in this context?

To allow a better understanding of the acquisition of the materials/texts used, it is worth highlighting that the research was made in the following sources of information: PubMed/MedLine and the website FurScience. The following terms were used as keywords/descriptors: subculture, fandom, social identity, anthropomorphism and stigma, in Portuguese and English, with the aim of seeking broader materials that would allow for better reflection on this subject. The construction of this article was carried out after the selection of bibliographic materials related to the aforementioned theme, their detailed reading to find the contents that corresponded to the researched attributes and the theoretical reflection on the theme, which served as a basis for the elaboration of the study. Thus, the main points of discussion were derived from the reflective analysis based on national and international literature, organized through two categories: “New identities: their expressions and social implications” and “Nursing care from the perspective of new identities”.

## DEVELOPMENT

### New Identities: Their Expressions and Social Implications

Identity is understood as a product of socialization based on contact with the world and the transformation of nature, legitimized in the dialogical relationship of the person/species/society triad. In social interactions, people develop identification with the groups to which they belong through the internalization of shared norms, values, and cultural traits, a process that contributes to the formation of self-concept and guides individual behavior. Such groups, in turn, outline their own subcultures, establish the constitutive references of their collective identity, and promote a sense of belonging among their members^(3,10)^. In this sense, individual identity is juxtaposed with social/cultural identity, conceived as the set formed by the individual’s self-concept, their group belongingness, and the value attributed to this belongingness^(2)^. Thus, human identity is polymorphic insofar as each person is unique and, at the same time, diverse, in their multiple forms of expression, within cultural plurality^(2,10)^.

For some social philosophers, the geek subculture is considered a super-subculture for three reasons. First, because it offers its followers two fundamental dimensions of identification – virtual time and digital space; second, because it proposes clear criteria of self-reflection for its participants and, finally, because it imposes strict demands on symbolic relationships, such as creativity, originality, and dedication to the cause, elements that favor the overcoming of its original fragmentary nature^(11)^. Even though their fandoms integrate people of different ages and generations, the geek subculture is essentially youthful worldwide, with the predominant age group being 16 to 29 years old, influenced by a lifestyle strongly linked to leisure and consumption^(12,13)^. The formation of cultural groups/subcultures has helped to demonstrate the need for many young people to seek to belong to something, and to produce new identities based on different cultural artifacts^(13)^.

There is a consensus regarding the importance of belonging for the construction of identity and for the psychological development of adolescents/young people. Authors argue that this sense of belonging can be obtained and reinforced by engaging in geek fandoms. Through the creation of videos and the participation on online discussions, young people can find a receptive audience for their writing or music and gain new skills to create and build. They can demonstrate their artistic skills in the form of photographs, crafts or drawings^(5,7)^. Therefore, the geek fandoms provide young people with the interaction in groups they identify with and develop a sense of pride for their contributions to these growing communities^(5,7,10)^.

Although the sense of identity and belongingness can be reaffirmed by integrating fandoms/communities of the geek subculture, two of them stand out for having a marked social stigma, even among the “geeks” themselves, the fandoms brony and furry^(7)^, which, even though they share an interest in anthropomorphic characters/animals, are distinct from each other^(14)^. It is due to this interest that the labels of “atypical”, “unconventional” and “non-human” identities appear in popular media in a distorted way.

The typical brony is a male teenager or young adult who is part of the fandom from the children’s cartoon television series My Little Pony: Friendship Is Magic. The “bronies” describe their admiration for the series as an appreciation for its aesthetic design and positive messages of friendship and acceptance. Some critics suggest that this represents a form of “neo-sincerity”: despite really liking these elements of the design, the “bronies” present a sense of postmodern irony and cynicism. They understand that the conventional society sees their admiration for the series in question as deviant and, therefore, they use irony as a form of protection against possible negative reactions. The term brony is a combination of “bro” (hypermasculinization of “brother”) and “pony”, a typically feminine symbol. The fusion between masculinity and feminine identity symbolizes the ironic stance of the fandom. The fanfics^*^ based on ponies are common, including clop-fic, which explores erotic narratives. Many fans also produce pony-inspired art, some with sexualized undertones. This content can strongly contribute to the negative view that a large part of the geek community and of the society in general presents about the “bronies”^(7)^.

The furry fandom, in turn, is associated with cosplay because they both involve the use of costumes, but their interests are different. People considered “furries” value anthropomorphic art and storytelling and express their connection with manga-style drawings of human-animal hybrids. Based on this connection, they create their own characters (fursonas)—symbolic and idealized representations of themselves, endowed with positive attributes such as sociability, extroversion, and self-confidence, and which serve as identity personifications within the fandom. They also deeply appreciate the creation of related fanfics and fursuits—costumes that materialize their fursonas and allow a deeper performative immersion into their animalized identity^(15)^.

The interaction among “furries” occurs both in the virtual environment, through chat rooms, discussion forums, and online games, in which anthropomorphic avatars represent the players, and in person, through conventions. There are events of this nature in large cities, specifically for “furries”, but they also participate in multigender geek conventions. The use of a fursuit in conventions is not a mandatory requirement to be considered a furry, since its production can take months and be expensive. As an affordable alternative, many fans wear animal ears, tails, and other accessories; some even choose not to dress up^(7,15,16,17)^. However, it is important to emphasize that the “furries” identify themselves **with** animals and not **as** animals^(15,18)^ and, therefore, they do not see or feel physical changes in their bodies, they do not move using all four limbs, they do not roar, nor do they ingest hay, grass or other types of food specific to non-human animals^(19)^.

“Geeks” from others fandoms, who make a greater distinction between their interests and their identity, consider the “furries” as extremes. In many communities, the marginalization of the most radical members manifests itself as a strategy to avoid the group being labeled or judged by outside observers, based on the behaviors and beliefs of this minority. A subgroup within the furry community, known as yiffies or plushies, allegedly engages in sexual activity while wearing fursuits. Although they represent a minority, the stigma associated with them is often generalized to the entire furry community^(7)^. In popular media, this prejudice is reinforced by portraying the “furries” as a fetishist fandom or as a group of people involved in sexually deviant behavior^(15,20)^.

Recent research has shown that non-sexual motivations are the most relevant to the furry social identity, especially the sense of belongingness and that, therefore, the “furries” can be more appropriately theorized as a fandom centered on media with sexual elements, rather than a sexual subculture with elements of fandom^(15,16,20)^.

Many of the negative stereotypes about the furry fandom have been empirically tested and found to be unfounded. One of them concerns a study^(20)^ that addressed the relationship between the furry community and zoophilia. According to this research, “furryism” has been conceptualized as a type of Class I Zoophilia, in which humans play the roles of non-human animals in sexual and non-sexual contexts. However, the conclusion indicates that most people with zoophilia were not furries and that although there was a correlation between the furry identity and some indicators of zoophilia, this relationship became statistically insignificant when the variable “self-identified zoophilia” was controlled. That is, the connection between being furry and having indicators of zoophilia only existed due to the overlap among those who already identified themselves as zoophiles. Therefore, the results of the study suggest that being furry, in itself, does not imply zoophilic tendencies and that any association between the two groups is explained only by the presence of people with zoophilia within the sample analyzed^(20)^.

Even if there are unusual sexual motivations and interests, this fact does not justify discrimination and stigmatization^(16)^. However, due to this possibility, the “furries” take different approaches to managing their identity among their peers. Some choose to keep their lives compartmentalized and do not discuss, outside the fandom, their involvement with it. Others express only a general connection to the geek subculture, focusing on interests like anime and manga to divert attention from their furry identity. Others, fewer in number, embrace it openly, promoting their playful and fun aspects by joking about the topic in conversations, or when going out with friends dressed up in costumes to unexpected places^(7)^.

Other emerging communities are the “otherkins” and the “therians”. The former are people who believe they are mentally or spiritually connected with non-human, often mystical, species such as dragons and minotaurs, or even extraterrestrial beings. The “therians” (short for therianthrope), in turn, believe in their deep mental or spiritual connection with real non-human animals, whether extant or extinct. Identification with an animal side or theriotype does not imply the existence of two distinct beings. A therianthrope is not a human animal on the one hand and a non-human animal on the other, as if they were two beings sharing a single body. In this case, the animal side is an indivisible part of the “self”, and there may be greater or lesser integration with it. Being a “therian” is described as a deep, personal and non-transferable experience, of a permanent nature, and not something circumstantial. In both groups, the connection to non-human species does not extend to physical form^(21)^.

Due to the different views “furries”, “otherkins”, and “therians” present themselves and among themselves, even if there is some overlap, the “furries” essentially constitute a fandom (fan community), while the “otherkins/therians” are communities based on beliefs and self-expression. This suggests that the main difference between the “furries” and the other two groups is not necessarily their appreciation for animals, but rather the degree to which they feel a deeper spiritual or identity connection with them^(22)^. However, researchers consider “bronies”, “otherkins”, “therians”, among others, as subgroups of the furry fandom. In their studies, these researchers found that the fandom itself points to the existence of “problematic furries” and controversial subgroups (such as those previously mentioned) and this perception generates prejudice, discrimination, intolerance, and bullying also within the fandom, besides the recognition that the bullying in fact occurs. Even if it is not physical, there is a significant social bullying (e.g., spreading rumors, practicing ostracism, among others), with most of the “furries” going through this kind of experience at some point in life. The reports of bullying occurred more frequently outside the fandom and were most pronounced between the ages of 11 and 18, a critical period for the formation of a person’s identity^(18,23)^.

There is a give and take scenario that sustains the vicious cycle of negative stereotypes and social stigma: on the one hand, out of fear of ridicule and other negative repercussions, “furries” prefer not to reveal their furry identity, especially those who are more involved in the fandom and who carry out professional activities or belong to families in which such disclosure could generate disapproval or conflict. This means that, the more strongly a person identifies as furry, the more likely to feel more harrassed by society for this reason. In contrast, precisely because many feel excluded and the target of provocations for being transgender or non-heterosexual, characteristics that, in themselves, are associated with a greater history of bullying, they identify themselves with a community of other self-proclaimed “outsiders” or “socially maladjusted.” This suggests that the involvement with the fandom can mitigate the negative impacts of bullying^(23)^.

In this context, the International Anthropomorphic Research Project (IARP), from the University of Waterloo (Ontario, Canada), has dedicated itself for the last two decades to research on fandoms, especially the furry fandom, and to the creation of the FurScience website, with the aim of demystifying it and combating misinformation. Recent episodes of false information have occurred in the United States about children dressed as animals, calling themselves “furries” and requiring sandboxes in schools instead of bathrooms^(23)^.

For IARP researchers, people in general have a tendency to think that there is “something wrong” with the “furries”, a perception reflected in media representations and in negative stereotypes about such fandom, insisting that its members need to be “explained” in clinical terms. Some people suggest the presence of psychological or developmental problems. Others venture situational explanations, such as a difficult, tumultuous childhood, without friends or marked by social difficulties. After all, there is consistent evidence that the bullying, stigma and the concealment of stigmatized identities can be especially detrimental to a person’s well-being. Therefore, it would be reasonable to expect that the “furries” showed signs of significantly compromised well-being. However, data from the literature suggest otherwise: there are no statistically significant differences in well-being, self-esteem, and life satisfaction compared to the general population^(15,18)^, besides the fact that the “furries” have demonstrated a more stable and coherent sense of identity than non-furries^(23,24)^.

Although they have more active fantasy lives, they are no more likely than other people to engage in pathological forms of fantasy (e.g., delusion, escapism, excessive fantasy); they tend to engage for healthy and beneficial reasons, including inspiring creativity, social interaction, and recreation. The researchers also highlight that, as a result of participating in a community that values tolerance, acceptance, and open-mindedness, interaction with the furry fandom is associated with global citizenship and a sense of responsibility of acting to improve the world (e.g., environmental concerns and valuing of diversity)^(15,18)^.

### Nursing Care from The Perspective of New Identities

When reflecting that the nursing care process has the human being as its main focus, it is undeniable that professional nursing conduct is “based on humanization and acceptance, without distinction of persons”. A greater understanding of people’s cultural essence will clarify the perception of who the patient/client/user is, minimizing potential barriers in the care relationship, particularly those that may emerge from stereotyping. Understanding the lifestyle, habits, and everything that surrounds people in their subcultures is the first step towards creating an effective therapeutic bond and providing humanized, quality care^(25)^.

Nurses and other nursing professionals who especially care for adolescents and young people require a practical understanding of these mostly hidden subcultures/communities. A young person’s interest in participating in a fandom may be met with familiar skepticism and distrust. Knowledge about these interests by nurses and the nursing team can help the young person participate safely and productively, providing them with the acceptance and support required for the exploration of their identity, which is fundamental for their psychological maturation^(7)^.

In Western cultures, it is quite common for sports fans to wear their team’s jersey to a special event like a game, or for a music fan to wear their favorite band’s t-shirt. The same goes for the members of the geek fandoms. However, the belief prevails that these people dress and behave this way all the time, on a daily basis, which is untrue^(15)^. Such attitudes cause strangeness, discomfort, or even a reaction of rejection in some people, for whom those who are “different” are “sick” or morally inadequate. This fulfills multiple protective functions, such as self-esteem preservation and sustainment of a positive self-image in the eyes of others, the perspectives of which may be challenging or divergent from one’s own^(23)^.

In this context, nursing care must first be guided at breaking the tendency towards “pathologization”. Despite the assumption implicit in stereotypes that only people with emotional distress or prone to “psychological problems” engage in geek activities or that such involvement results in these conditions, there is no evidence to support this assumption^(5,8,20,23)^. Conversely, studies report that the “geeks” demonstrate greater civic engagement and do not exhibit deficits in belonging, social network size, or future orientation. Therefore, there is a consensus that they are different, but not dysfunctional^(5,7,8,23)^. Hence, they require greater attention from health professionals, especially nursing, an occupation that is directly linked to the population^(25)^. This understanding allows extricating from the prejudice and negative stereotypes that surround geek fandoms.

Because it is a subculture that shares its interests almost always through online forums, social media networks and platforms, the use of digital technologies can allow the approach to this specific clientele. Furthermore, in virtual environments, clashes that result in bullying/cyberbullying are more frequent, since social media platforms are not able to monitor all messages posted by their users. The supposed anonymity of social media causes some users to spread hate or avoid civilized behavior, with the false perception that there are no real consequences for these acts^(7)^. The incorporation of these technologies as channels for active and qualified listening is seen as an interesting strategy for nursing care, as it allows professionals to quickly identify people who need support, as well as possible interventions and referrals.

Another aspect to be considered in care concerns the inclusion of leisure to establish closer and more embracing relationships with people, respecting their subjectivity and cultural identity. Leisure activities are designed to meet personal needs and produce different effects depending on each person’s values and objectives. The essential thing in this practice is to contribute to the development of individual potential and promote well-being^(26)^. The geek activities fulfill this role by transcending mere entertainment, as for their followers, they represent significant mechanisms of socialization and identity construction. They also boost creativity and act as relevant sources of emotional resilience^(24)^, thus contributing to the consolidation of interpersonal trust^(15)^. Therefore, it is up to nursing professionals to encourage leisure activities that promote autonomy and personal satisfaction. This approach is in line with the literature on coping strategies through leisure (leisure coping)^(5)^.

Promoting embracing and symbolically inclusive clinical environments is another tool for cultural engagement, essential for breaking down stigma in the community and within healthcare services. This can range from making the language used in consultations more flexible to displaying visual cues that demonstrate familiarity and respect for the geek subculture—such as posters with popular characters and the use of interactive or gamification technologies. This environment can reinforce the therapeutic bond, promote communication, and minimize cultural barriers that hinder the professional-patient relationship^(27)^. Regarding this last aspect, demonstrating genuine curiosity regarding the person’s interests, using elements of their subculture as facilitators in building this relationship, as well as understanding that they can use references from games, films or characters as emotional coping mechanisms, contributes to more empathetic and individualized professional behaviors.

It is also the nurse’s responsibility to assume their role as educator, providing evidence-based information about subcultures, fandoms and their respective identities, activities, and interests. Promoting discussion groups, lectures, awareness campaigns, and supporting initiatives that promote equity and respect for diversity are essential strategies for reducing social prejudice^(28)^. Accordingly, health care professionals have to acquire and/or improve their cultural competence, as it is essential that such competence be incorporated in a more in-depth and systematic manner into their academic training, to prepare them for the job market with a broader understanding of cultural diversity. This way, the discomfort that the contact with different realities generates, to the point of compromising professional practice, is prevented. Moreover, it is essential to promote ongoing in-service education throughout the career path, aiming to improve skills for interacting with different cultural contexts. This continuous investment allows nurses, technicians, and nursing assistants, even when faced with challenges and barriers, to be able to develop effective solutions that are sensitive to cultural/subcultural specificities^(25)^.

A starting point for fully developing cultural competence is to seize the assumptions of Transcultural Nursing through Madeleine Leininger’s Theory of Diversity and Universality of Cultural Care (TDUCC). This theory aims to provide a form of care that is sensitive and based on the needs of the person, their family and the cultural groups to which they belong. To this end, it is essential to know these groups’ peculiarities to find similarities and differences in relation to care and health behaviors. This knowledge allows us to develop culturally appropriate care consistent with the specific needs of a given population. Nurses, nursing technicians and assistants with cultural competence efficiently improve communication skills, establish horizontal relationships with those they care for in acting, listening and thinking, and awaken people’s engagement in health care by making them believe that they are valued and respected in their way of life^(29)^.

Knowing the geek subculture, as proposed in this theoretical-reflective essay, favors an approach to TDUCC and can be an initial step in the development of cultural competence, so that it clearly reflects on ethical competence for a more humanistic approach to stigmatized people^(28)^. Professional behavior in nursing, as in other professions, is an expression of the ethical values that support the nursing profession, given that their attitudes and choices directly impact people’s lives and well-being, in addition to influencing the quality of care, the work environment and the relationship of trust between the team and those under their care. In this regard, the ethical competence of these professionals is manifested in the ability to analyze situations and act according to the principles that ensure the people’s protection, respect for the rights, dignity, and integrity^(30)^.

Another aspect observed in the literature, related to stigmatized people, is that the relationships between the person and the family and between the person and the health professional, can be overprotective or neglected. Both circumstances can generate insecurity/fear due to a lack of confidence in the provision of support^(28)^. Research on the furry fandom, for example, shows that the “furries”, when seeking advice for physical or psychological conditions, had their problems ignored by several health professionals, who, in fact, focused on the fact that “being a furry” was the problem^(18)^. It is in this scenario that the nurse’s cultural and ethical skills make a difference, so that the professional will demonstrate the skills to intervene and create spaces of care, in which sensitivity, acceptance, qualified listening and openness to dialogue predominate, in a respectful and empathetic communication process. Consequently, those who feel stigmatized and their families will be able to talk, express their fears or doubts, and propose other perspectives for society and for themselves^(25,28,31)^.

Nursing care is essentially ethical insofar as actions are guided by integrity and decisions made prioritize humanitarian aspects and the well-being of those being cared for, seeking their best interests. However, nurses’ daily professional practice is permeated by conflicting situations and difficulties, especially work overload, scarcity of resources and lack of institutional support, which can limit ethical practice. In this sense, continuing education in service, as already mentioned, has been recognized as a valuable resource for professional improvement, both from a cultural and ethical point of view. Nurses and nursing teams who participate in continuing education feel more confident and capable of working in “problematic” or culturally diverse contexts, which has a positive impact on the satisfaction of those receiving care and on the retention of professionals in healthcare institutions^(30)^.

In addition to theoretical concepts, the considerations highlighted here in a reflective manner had the purpose of deconstructing the labels of new identities as being “atypical”, “unconventional” or “non-human”, and of awakening nursing and other health professionals to the improvement of cultural and ethical skills, to redefine care for people stigmatized by their activities, interests, and forms of social interaction. It is also important to emphasize that social stigma has implications for the health of those who feel stigmatized, demanding even more complex interpersonal skills from professionals for comprehensive care. Thus, self-knowledge and personal willingness to change constitute the initial steps towards promoting health, as well as towards a relationship of respect and trust between the caregiver and the person being cared for^(31)^.

By embracing holistic care, it is possible to reflect and understand that the concepts of normality and stigmatization do not refer to people themselves, but to perspectives constructed in specific social contexts. These understandings emerge in mixed interactions, being shaped by the perception of non-compliance with social norms, which influence the dynamics of interactions, including those with health professionals, such as nurses and nursing teams. Therefore, to change the reality of stigma, it is imperative to focus not on differences, but on what makes us similar: we are all human beings^(31)^.

## Limitations And Contributions Of The Study

The limitations refer to the scarcity of studies on the topic, especially regarding nursing care in the context of the identities presented, which hindered a more in-depth analysis. Additionaly, the use of grey literature was necessary as it contains some fundamental aspects of the proposed theme. Another limitation concerns the method chosen, which does not allow for classifying the strength of the evidence identified.

In contrast, its main contribution lies in reducing the knowledge gap on the topic, especially in the health field, by clarifying subcultural peculiarities that, if ignored or minimized, can result in failures in therapeutic communication, resistance to or dissatisfaction with care, a feeling of not belonging in health care spaces, and inequities in access to and quality of care. Furthermore, given the advances in digital transformation in healthcare, this essay aims to problematize the preparation of nursing professionals to interact effectively with subcultures deeply integrated into virtual environments and online communities. This new digital context challenges traditional care models, requiring professionals not only to have technical, but also cultural competence, and openness to new forms of care mediation.

## CONCLUSION

This theoretical-reflective essay discussed the new identities, their expressions and motivations, and presents some insights about nursing care in this context. It is believed that the discussions arising from reflection can allow health professionals, especially nurses and nursing teams, to demystify virtual subcultures/communities, to overcome possible barriers that may arise in care relationships, due to stigmatization. Other contributions of this essay refer to the promotion of debates during the professional training course in health regarding the conception of beliefs, stereotypes, prejudices, and the factors that favor their deconstruction. Finally, we hope to strengthen nursing’s cultural and ethical competencies to redefine the process of caring for stigmatized people.

## Data Availability

The database supporting this article is not publicy available.

## Footnotes

* Fanfic is a term derived from the English “fan fiction”. These are fictional stories created by fans, based on the plot and characters of films, series, books or games^(10)^
