# Supplementary material for: Nursing care from the perspective of subcultural identities: a reflective essay
Source: Rev Esc Enferm USP. 2025 Oct 20;59:e20250140. doi: 10.1590/1980-220X-REEUSP-2025-0140en (PMC12543436; doi:10.1590/1980-220X-REEUSP-2025-0140en)
Supplement: Translation

# Translation: O cuidado de enfermagem na perspectiva das identidades subculturais: um ensaio reflexivo

**DOI:** 10.1590/1980-220X-REEUSP-2025-0140pt

**Authors:** Lilian Cristiane Gomes, Maria Lúcia do Carmo Cruz Robazzi, Sérgio Valverde Marques dos Santos, Fábio de Souza Terra, Mônica La Salette da Costa Godinho, Cristiane Aparecida Silveira, Ana Kelley de Rezende, João Vitor Andrade, Namie Okino Sawada

**Affiliations:** 1 Universidade Federal de Alfenas, Escola de Enfermagem, Alfenas, MG, Brasil.

**Keywords:** Identificação Social, Diversidade Cultural, Estereotipagem, Cuidados de Enfermagem, Competência Cultural

## Abstract

Este estudo teórico-reflexivo investigou a Diversidade Identitária e o Cuidado de Enfermagem, com base em materiais selecionados no PubMed/MedLine e FurScience, proporcionando uma reflexão crítica sobre o tema. Foram desenvolvidas duas categorias: “Novas identidades: suas expressões e implicações sociais”, que aborda a construção de identidades sociais e subculturais em grupos com interesses comuns e como essas identidades influenciam a sociedade e “O cuidado de Enfermagem na perspectiva das novas identidades”, que destaca a importância do conhecimento sobre as subculturas e identidades sociais no cuidado, especialmente com adolescentes e jovens. Esse entendimento favorece a humanização, fortalece o vínculo terapêutico e minimiza barreiras na assistência de enfermagem. As novas identidades são vistas como construções socioculturais dinâmicas, cuja reflexão teórica ressalta a importância de desmistificar estigmas e promover um cuidado humanizado. Além disso, enfatiza as competências cultural e ética da enfermagem na ressignificação do cuidado às pessoas estigmatizadas, incentivando debates na formação profissional em saúde.

## INTRODUÇÃO

Em um mundo globalizado, permeado de inovações e avanços tecnológicos cada vez mais rápidos e significativos^(1)^, despontam novas formas de socialização e de agregação social, as quais, por sua vez, produzem as suas próprias culturas ou subculturas^(2,3)^. O termo “subcultura” refere-se a valores, crenças, atitudes, identidade e estilos de vida de um grupo dentro da sociedade^(4)^. Como exemplo, tem-se as subculturas *nerd* e *geek*, nas quais os adeptos são pessoas excepcionalmente envolvidas ou interessadas, respectivamente, em tecnologias/ciências exatas e cultura *pop/*mundos inventados (histórias em quadrinhos, animes e mangás, *cosplay*, entre outros), sendo especialistas nessas áreas. A subcultura *geek*, particularmente, tem ganhado grande popularidade e nela, cada área de interesse constitui um “*fandom*”, ou seja, uma comunidade de fãs ou simpatizantes^(5–8)^.

Antes da era digital, aqueles que se identificavam como “*geeks*” aparentavam-se isolados, tanto em seus interesses, quanto de seus pares. Isso porque muitas atividades *geek* (como ler, escrever e jogar) eram consideradas inerentemente “menos sociais” pois, na maioria das vezes, eram realizadas a sós. Mesmo os “*geeks*” que viviam em grandes cidades enfrentavam dificuldades para encontrar e conectar-se com outras pessoas que participavam dos mesmos *fandom*s/comunidades^(7)^. O apego e a realização dessas atividades eram vistos, de certa forma, como um comportamento antissocial ou como um sinal de indiferença às interações sociais, resultando em estereótipos negativos^(5,7,8)^. O advento da Internet, entretanto, oportunizou às pessoas conectarem-se livremente, sem restrições de tempo, distância, gênero ou idade. Tal como na vida real, aquelas com interesses em comum começaram a se reunir em espaços virtuais para socializar. Em geral, adolescentes e jovens adaptaram-se mais rapidamente ao novo cenário, auxiliados por sua capacidade de assimilar tecnologia com esforço mínimo. Assim, abriu-se um leque de possibilidades para os jovens escolherem ao definirem-se e apresentarem-se ao mundo^(7)^.

Ao refletir sobre o fato de que os interesses e gostos são mutáveis, torna-se compreensível que atividades anteriormente maculadas por demasiado estigma social podem, agora, conquistar capital social on-line^(7)^. Esse fato é tão genuíno que, desde a última década, as atividades *geek* vêm se tornando sucessos de bilheteria, indústrias multibilionárias e uma subcultura de grande alcance, com suas próprias marcas, tendências de moda e celebridades. Faz-se necessário refletir, também, que apesar de comumente associada à recreação ou ao entretenimento, a subcultura *geek* constitui um componente essencial da vida de muitos entusiastas por influenciar suas experiências emocionais, comportamentais e até suas percepções sobre saúde e autocuidado^(5)^.

É nesse contexto subcultural que a adoção de novas identidades, denominadas “não convencionais”, “atípicas”, ou “não-humanas”, emerge extensivamente^(9)^. E, embora o fenômeno da globalização e a profusão das tecnologias digitais tenham fomentado um maior movimento de ideias, bem como uma maior circulação de pessoas e de informações^(1)^, pouco ainda se sabe sobre esse universo “neo-identitário”. Cumpre ressaltar que o termo “novas identidades” aqui empregado não se refere à sua cronologia/novidade temporal, mas à escassa discussão que suscita na literatura científica na área da saúde, o que contribui para perdurar o preconceito, os estereótipos e a desinformação. Tal situação, além de levantar questionamentos relevantes, exige a atenção dos profissionais da saúde, sobretudo da enfermagem, enquanto profissão voltada ao cuidado integral do ser humano e, frequentemente, responsável pelo acolhimento inicial nos serviços de saúde. Considerando a especificidade de suas funções, é imprescindível que esses profissionais estejam atentos a essas singularidades culturais para reconhecer, compreender e respeitar práticas e identidades de subculturas digitais e midiáticas, como a *geek*, a fim de estabelecer vínculos terapêuticos sustentados em uma perspectiva empática e compreensiva com quem cuida.

Dessa forma, este estudo pretende não somente clarificar as definições subjacentes às novas identidades e suas implicações sociais, mas, principalmente, fornecer um arcabouço teórico-reflexivo que possa auxiliar enfermeiros(as) e equipes de enfermagem na abordagem e na prestação de cuidados às pessoas, bem como no apoio e esclarecimento às famílias, no intuito de prevenir e/ou minimizar o viés da estigmatização e possíveis formas de sofrimento psicoemocional. Assim, o objetivo do presente estudo é discorrer sobre as novas identidades, as razões que as sustentam e suas repercussões para o cuidado de enfermagem.

## MÉTODO

Trata-se de um ensaio teórico-reflexivo, elaborado por meio de uma revisão da literatura, de forma narrativa e a partir das seguintes inquietações: quais são as novas identidades e o que as influenciam e/ou motivam? Que reflexões emergem sobre o cuidado de enfermagem nesse contexto?

Para permitir uma melhor compreensão sobre a aquisição dos materiais/textos utilizados, destaca-se que a pesquisa ocorreu nas seguintes fontes de informação: PubMed/MedLine e o website *FurScience*. Foram utilizados como palavras-chaves/descritores os termos: subcultura, *fandom*, identidade social, antropomorfismo e estigma, nos idiomas português e inglês, com o intuito de buscar materiais mais amplos e que permitissem uma melhor reflexão sobre este assunto. A construção deste artigo conduziu-se após a seleção dos materiais bibliográficos relacionados com a referida temática, a sua leitura minuciosa a fim de identificar os conteúdos que correspondiam aos atributos pesquisados e a reflexão teórica sobre o tema, que serviram de base para a elaboração do estudo. Com isso, os principais pontos de discussão foram decorrentes da análise reflexiva fundamentada na literatura nacional e internacional, organizados por meio de duas categorias: “Novas identidades: suas expressões e implicações sociais” e “O cuidado de Enfermagem na perspectiva das novas identidades”.

## DESENVOLVIMENTO

### Novas Identidades: Suas Expressões e Implicações Sociais

A identidade é entendida como um produto da socialização a partir do contato com o mundo e da transformação da natureza, legitimada na relação dialógica da tríade pessoa/espécie/sociedade. Nas interações sociais, as pessoas desenvolvem identificação com os grupos aos quais pertencem mediante a internalização de normas, valores e traços culturais compartilhados, processo que contribui para a formação do autoconceito e orienta as condutas individuais. Tais grupos, por sua vez, delineiam subculturas próprias, estabelecem as referências constitutivas de sua identidade coletiva e promovem o senso de pertencimento entre seus membros^(3,10)^. Nesse sentido, a identidade individual justapõe-se à social/cultural, concebida como o conjunto formado pelo autoconceito do indivíduo, sua pertença grupal e a valoração atribuída a esta pertença^(2)^. Assim, a identidade humana apresenta-se polimorfa na medida em que cada pessoa é singular e, ao mesmo tempo diversa, em suas múltiplas formas de expressão, dentro da pluralidade cultural^(2,10)^.

Para alguns filósofos sociais, a subcultura ^(13)^.

Há um consenso quanto à importância do pertencimento para a construção da identidade e para o desenvolvimento psicológico dos adolescentes/jovens. Autores advogam que esse senso de pertença pode ser obtido e reforçado pelo engajamento nos *fandoms geek*. Por meio da criação de vídeos e da participação em discussões on-line, os jovens podem encontrar um público receptivo à sua escrita ou música e adquirirem novas competências para criar e construir. Podem demonstrar as suas habilidades artísticas na forma de fotografias, artesanato ou desenhos^(5,7)^. Portanto, os *fandoms geek* propiciam aos jovens a interação em grupos com os quais se identificam e desenvolvem um senso de orgulho pelas suas contribuições nessas comunidades em crescimento^(5,7,10)^.

Embora o senso de identidade e de pertença possam ser reafirmados ao integrar *fandom*s/comunidades da subcultura *geek*, dois deles destacam-se por possuir um marcante estigma social, inclusive entre os próprios “*geeks*”, sendo os *fandom*s *brony* e *furry* ^(7)^, os quais, mesmo compartilhando um interesse por personagens/animais antropomórficos, são distintos entre si^(14)^. É devido a esse interesse que surgem, nas mídias populares e de forma distorcida, os rótulos de “identidades atípicas”, “não convencionais” e “não-humanas”.

O típico *brony* é um adolescente ou jovem adulto do sexo masculino que integra o *fandom* da série televisiva de desenho infantil *My Little Pony: Friendship Is Magic*. Os “*bronies*” descrevem sua admiração pela série como uma apreciação do design estético e das mensagens positivas de amizade e aceitação. Alguns críticos sugerem que isso representa uma forma de “neo-sinceridade”: apesar de realmente gostarem desses elementos do desenho, os “*bronies*” apresentam um senso de ironia pós-moderna e cinismo. Eles compreendem que a sociedade convencional enxerga sua admiração pela referida série como algo desviante e, portanto, utilizam a ironia como uma forma de proteção contra possíveis reações negativas. O termo *brony* é uma junção de “*bro*” (hipermasculinização de “*brother*”) e “*pony*”, símbolo tipicamente feminino. A fusão entre masculinidade e identidade feminina simboliza a postura irônica do *fandom*. As *fanfics* ^*^ baseadas nos pôneis são comuns, incluindo *clop-fic*, que explora narrativas eróticas. Muitos fãs também produzem arte inspirada nos pôneis, algumas com tons sexualizados. Esse conteúdo pode contribuir fortemente para a visão negativa que grande parte da comunidade *geek* e da sociedade em geral apresenta sobre os “*bronies*”^(7)^.

O *fandom furry* (do inglês, “peludo”), por sua vez, é associado ao *cosplay* porque ambos envolvem o uso de fantasias, mas os seus interesses são diferentes. As pessoas consideradas “furries” valorizam a arte e a narrativa antropomórficas e expressam sua conexão com desenhos de híbridos humano-animais em estilo mangá. A partir dessa conexão, criam seus os próprios personagens (*fursonas*)—representações simbólicas e idealizadas de si mesmos, dotadas de atributos positivos como sociabilidade, extroversão e autoconfiança e que servem como personificações identitárias dentro do *fandom*. Também apreciam intensamente a criação de *fanfics* relacionadas e de *fursuits*—fantasias que materializam suas *fursonas* e permitem uma imersão performática mais profunda em sua identidade animalizada^(15)^.

A interação entre os “*furries*” ocorre tanto no ambiente virtual, por meio de salas de bate-papo, fóruns de discussão e jogos on-line, nos quais avatares antropomórficos representam os jogadores, quanto presencialmente, por meio das convenções. Há eventos dessa natureza nas grandes cidades, específicos para os “*furries*”, porém eles também participam de convenções *geek* multigênero. O uso de um *fursuit* em convenções não é um requisito obrigatório para ser considerado *furry*, visto que sua confecção pode levar meses e ser onerosa. Como alternativa acessível, muitos adeptos usam orelhas, caudas de animais e outros acessórios; alguns, ainda, optam por não se fantasiar^(7,15–17)^. Todavia, é importante enfatizar que os “*furries*” identificam-se **com** animais e não **como** animais^(15,18)^ e, portanto, não veem nem sentem mudanças físicas em seus corpos, não se locomovem usando os quatro membros, não bramem e nem, tampouco, ingerem feno, grama ou outro tipo de alimento específico para animais não humanos^(19)^.

“*Geeks*” de outros *fandom*s, que fazem uma distinção maior entre seus interesses e sua identidade, consideram os “*furries*” como extremos. Em muitas comunidades, a marginalização dos membros mais radicais manifesta-se como uma estratégia para evitar que o grupo seja rotulado ou julgado por observadores externos, com base nos comportamentos e crenças dessa minoria. Um subgrupo dentro da comunidade *furry*, conhecido como *yiffies* ou *plushies*, supostamente participa de atividades sexuais enquanto usa *fursuits*. Embora representem uma minoria, o estigma associado a eles, muitas vezes, é generalizado para toda a comunidade *furry* ^(7)^. Na mídia popular, esse preconceito é reforçado ao retratar os “*furries*” como um *fandom* fetichista ou como um grupo de pessoas envolvidas em comportamentos sexualmente desviantes^(15,20)^.

Pesquisas recentes têm evidenciado que as motivações não sexuais são as de maior relevância para a identidade social *furry*, principalmente o senso de pertencimento e que, portanto, os “*furries*” podem ser mais apropriadamente teorizados como um *fandom* centrado em mídia com elementos sexuais, do que uma subcultura sexual com elementos de *fandom* ^(15,16,20)^.

Muitos dos estereótipos negativos sobre o ^(20)^.

Ainda que haja motivação e interesses sexuais incomuns, tal fato não justifica a discriminação e a estigmatização^(16)^. Porém, devido a essa possibilidade, os “^(7)^.

Outras comunidades emergentes são os “*otherkins*” e os “*therians*”. Os primeiros são pessoas que acreditam ser mental ou espiritualmente conectadas com espécies não humanas, muitas vezes místicas, como dragões e minotauros ou, até mesmo, aos seres extraterrestres. Já os “*therians*” (abreviação de *therianthrope*) acreditam em sua conexão mental ou espiritual profunda com animais reais não-humanos, sejam eles existentes ou extintos. A identificação com um lado animal ou teriotipo, não implica a existência de dois seres distintos. Um teriantropo não é um animal humano por um lado e um animal não humano por outro, como se fossem dois seres compartilhando um único corpo. Nesse caso, o lado animal faz parte indivisível de seu “eu”, podendo haver maior ou menor integração com ele. Ser “*therian*” é descrito como uma experiência profunda, pessoal e intransferível, de caráter permanente, e não algo circunstancial. Em ambos os grupos, a conexão com as espécies não-humanas não se estende à forma física^(21)^.

Devido às diferentes visões que os “*furries*”, “*otherkins*” e “*therians*” apresentam de si e entre si, ainda que haja alguma sobreposição, os “*furries*” constituem, essencialmente, um *fandom* (comunidade de fãs), enquanto os “*otherkins/therians*” são comunidades fundamentadas em crenças e autoexpressão. Isso sugere que a principal diferença entre os “*furries*” e os outros dois grupos não está necessariamente no apreço pelos animais, mas sim, no grau em que sentem uma conexão espiritual ou identitária mais profunda com eles^(22)^. Entretanto, pesquisadores consideram “*bronies*”, “*otherkins*”, “*therians*”, entre outros, como subgrupos do *fandom furry*. Em seus estudos, esses pesquisadores encontraram que o próprio *fandom* aponta a existência de “*furries* problemáticos” e subgrupos controversos (tais como os anteriormente citados) e essa percepção gera preconceito, discriminação, intolerância e *bullying* também dentro do *fandom*, além do reconhecimento de que o *bullying* de fato ocorre. Mesmo que não seja físico, há um *bullying* social significativo (por exemplo, espalhar rumores, praticar ostracismo, entre outros), sendo que a maioria dos “*furries*” passa por esse tipo de experiência em algum momento da vida. Os relatos de *bullying* ocorreram com maior frequência fora do *fandom* e foram mais acentuados entre os 11 e 18 anos, um período crítico para a formação da identidade de uma pessoa^(18,23)^.

Há uma via de mão dupla que sustenta o ciclo vicioso de estereótipos negativos e estigma social: de um lado, pelo temor da ridicularização e de outras repercussões negativas, os “^(23)^.

Mediante esse contexto, o *International Anthropomorphic Research Project* (IARP), da Universidade de Waterloo (Ontário, Canadá), tem se dedicado nas duas últimas décadas, às pesquisas sobre *fandoms*, especialmente o *fandom furry* e à criação do *website FurScience*, com o objetivo de desmistificá-lo e combater a desinformação. Episódios recentes de informações falsas ocorreram nos Estados Unidos acerca de crianças vestidas de animais, chamando-se de “*furries*” e exigindo, nas escolas, caixas de areia ao invés de banheiros^(23)^.

Para os pesquisadores do IARP, as pessoas em geral têm a tendência de pensar que há “algo de errado” com os “*furries*”, uma percepção refletida nas representações da mídia e nos estereótipos negativos sobre tal *fandom*, insistindo que os seus integrantes precisam ser “explicados” em termos clínicos. Algumas pessoas sugerem a presença de problemas psicológicos ou de desenvolvimento. Outras arriscam explicações situacionais, como uma infância difícil, tumultuada, sem amigos ou marcada por dificuldades sociais. Afinal, há evidências consistentes de que o *bullying*, o estigma e a ocultação de identidades estigmatizadas podem ser especialmente prejudiciais ao bem-estar de uma pessoa. Portanto, seria razoável esperar que os “*furries*” apresentassem sinais de bem-estar significativamente comprometidos. Contudo, os dados da literatura sugerem o contrário: não há diferenças estatisticamente significantes quanto ao bem-estar, autoestima e satisfação com a vida na comparação com a população geral^(15,18)^, além do fato de que os “*furries*” têm demonstrado um senso de identidade mais estável e coerente do que os não-*furries* ^(23,24)^.

Embora tenham vidas de fantasia mais ativas, não são mais propensos do que as outras pessoas a envolverem-se em formas patológicas de fantasia (por exemplo, ilusão, escapismo, fantasia excessiva); a tendência do envolvimento é por razões saudáveis e benéficas, que incluem a inspiração à criatividade, interação social e recreação. Os pesquisadores ainda destacam que, como resultado da participação em uma comunidade que valoriza a tolerância, a aceitação e a mente aberta, a interação com o *fandom furry* é associada à cidadania global e a um senso de responsabilidade de agir para melhorar o mundo (por exemplo, preocupações ambientais e valorização da diversidade)^(15,18)^.

### O Cuidado de Enfermagem na Perspectiva das Novas Identidades

Ao refletir que o processo de cuidar em enfermagem tem como principal enfoque o ser humano, é inegável que a conduta profissional de enfermagem seja “pautada na humanização e no acolhimento, sem acepção de pessoas”. Um maior entendimento sobre a essência cultural das pessoas irá clarificar a percepção de quem é o paciente/cliente/usuário, minimizando eventuais barreiras na relação do cuidar, particularmente aquelas que podem emergir da estereotipização. Conhecer o estilo de vida, os costumes e tudo que cerca as pessoas em suas subculturas constitui-se no primeiro passo para a criação de um vínculo terapêutico efetivo e a prestação de um cuidado humanizado e de qualidade^(25)^.

Enfermeiros(as) e demais profissionais de enfermagem que assistem especialmente aos adolescentes e jovens requerem uma compreensão prática dessas subculturas/comunidades, em sua maioria, ocultas. O interesse de um jovem em participar de um ^(7)^.

Nas culturas ocidentais, é bastante comum que admiradores de esportes vistam a camisa de seu time em um evento especial como um jogo, ou que um fã de música traje a camiseta da sua banda favorita. O mesmo ocorre com os membros dos *fandoms geek,* porém, prevalece a crença de que essas pessoas vestem-se e comportam-se dessa maneira o tempo todo, cotidianamente, o que é uma inverdade^(15)^. Tais atitudes causam estranheza, incômodo/desconforto, ou até mesmo uma reação de rejeição em algumas pessoas, para as quais os “diferentes” são “doentes” ou moralmente inadequados. Isso cumpre múltiplas funções protetoras, como a preservação da autoestima e a sustentação de uma autoimagem positiva perante os outros, cujas perspectivas podem ser desafiadoras ou divergentes às suas próprias^(23)^.

Nesse contexto, o cuidado de enfermagem deve se orientar, primeiramente, a romper com a tendência à “patologização”. Apesar da suposição implícita nos estereótipos de que somente pessoas com sofrimento emocional ou propensas aos “problemas psíquicos” envolvem-se em atividades *geek* ou que tal envolvimento resulta nessas condições, não há evidências que sustentem essa suposição^(5,8,20,23)^. Ao contrário, estudos reportam que os “*geeks*” demonstram maior engajamento cívico e não apresentam déficits em pertencimento, tamanho da rede social ou orientação para o futuro. Assim, há um consenso de que eles são diferentes, mas não disfuncionais^(5,7,8,23)^, por isso, requerem uma maior atenção dos profissionais de saúde, principalmente da enfermagem, profissão que está diretamente ligada à população^(25)^. Essa compreensão possibilita desvencilhar-se do preconceito e dos estereótipos negativos que cercam os *fandoms geeks*.

Por ser uma subcultura que compartilha seus interesses quase sempre por meio de fóruns on-line*,* redes e plataformas de mídia social, o uso de tecnologias digitais pode possibilitar a aproximação com essa clientela específica. Além disso, em ambientes virtuais, os embates que resultam em *bullying/cyberbullying* são mais frequentes, uma vez que as plataformas de mídia social não conseguem monitorar todas as mensagens postadas por seus usuários. O suposto anonimato das redes sociais faz com que alguns usuários disseminem o ódio ou evitem comportamentos civilizados, com a falsa percepção de que não existem consequências reais para esses atos^(7)^. A incorporação dessas tecnologias como canais de escuta ativa e qualificada é vista como uma estratégia interessante para o cuidado de enfermagem, pois permite aos profissionais identificar, com celeridade, pessoas que necessitam de apoio, bem como possíveis intervenções e encaminhamentos.

Outro aspecto a ser considerado no cuidado diz respeito à inclusão do lazer para estabelecer relações mais próximas e acolhedoras com as pessoas, respeitando a sua subjetividade e a identidade cultural. As atividades de lazer propõem-se a atender às necessidades pessoais e produzem efeitos distintos conforme os valores e objetivos de cada pessoa. O essencial nessa prática é contribuir para o desenvolvimento das potencialidades individuais e promover o bem-estar^(26)^. As atividades *geek* cumprem esse papel ao transcederem o mero entretenimento, pois para os seus adeptos, representam mecanismos significativos de socialização e construção identitária. Também impulsionam a criatividade e atuam como fontes relevantes de resiliência emocional^(24)^, contribuindo assim, para a consolidação da confiança interpessoal^(15)^. Desse modo, cabe aos profissionais de enfermagem estimular as atividades de lazer promotoras de autonomia e satisfação pessoal. Essa abordagem está em consonância com a literatura sobre estratégias de enfrentamento por meio do lazer (*leisure coping*)^(5)^.

A promoção de ambientes clínicos acolhedores e simbolicamente inclusivos é outra ferramenta de aproximação cultural, imprescindível para quebrar o estigma na comunidade e dentro dos serviços de saúde. Isso pode incluir desde a flexibilização da linguagem utilizada nos atendimentos até a exibição de sinais visuais que demonstrem familiaridade e respeito com a subcultura *geek*—como cartazes com personagens populares e o uso de tecnologias interativas ou de gamificação. Essa ambientação pode reforçar o vínculo terapêutico, favorecer a comunicação e minimizar barreiras culturais que dificultem o relacionamento profissional-paciente^(27)^. Quanto a esse último aspecto, demonstrar curiosidade genuína em relação aos interesses da pessoa, empregando os elementos de sua subcultura como facilitadores na construção dessa relação, bem como compreender que ela possa utilizar referências de jogos, filmes ou personagens como mecanismos de enfrentamento emocional, contribui para condutas profissionais mais empáticas e individualizadas.

Também é responsabilidade do(a) enfermeiro(a) assumir o seu papel de educador(a), fornecendo informações baseadas em evidências sobre subculturas, *fandom*s e suas respectivas identidades, atividades e interesses. Fomentar rodas de conversa, palestras, campanhas de conscientização e apoiar iniciativas que promovam a equidade e o respeito à diversidade, são estratégias indispensáveis para reduzir o preconceito social^(28)^. Para tanto, torna-se necessária a aquisição e/ou o aprimoramento da competência cultural por parte desses profissionais de saúde, pois é fundamental que tal competência seja incorporada de maneira mais aprofundada e sistemática na sua formação acadêmica, de modo a prepará-los para o mercado de trabalho com uma compreensão ampliada sobre a diversidade cultural. Dessa forma, evita-se que o contato com realidades distintas gere desconforto a ponto de comprometer a prática profissional. Além disso, é essencial promover a educação permanente em serviço ao longo da trajetória laboral, visando aprimorar as habilidades de interação com diferentes contextos culturais. Esse investimento contínuo possibilita que, mesmo diante de desafios e barreiras, os(as) enfermeiros(as), técnicos(as) e auxiliares de enfermagem sejam capazes de desenvolver soluções eficazes e sensíveis às especificidades culturais/subculturais^(25)^.

Um ponto de partida para desenvolver plenamente a competência cultural é apropriar-se dos pressupostos da Enfermagem Transcultural por meio da Teoria da Diversidade e Universalidade do Cuidado Cultural (TDUCC), de Madeleine Leininger. Essa teoria objetiva prover uma forma de cuidado que seja sensível e fundamentada nas necessidades da pessoa, de sua família e dos grupos culturais aos quais pertence. Para isso, é essencial conhecer as peculiaridades desses grupos, a fim de identificar semelhanças e diferenças em relação ao cuidar e aos comportamentos de saúde. Esse conhecimento permite desenvolver um cuidado culturalmente adequado e coerente com as necessidades específicas de determinada população. Enfermeiros(as), técnicos(as) e auxiliares de enfermagem dotados(as) de competência cultural aperfeiçoam com eficiência a capacidade de comunicação, estabelecem relações horizontalizadas com quem cuida no agir, no ouvir e no pensar e despertam o engajamento das pessoas nos cuidados de saúde por fazê-las acreditar que são valorizadas e respeitadas em seu modo de viver^(29)^.

Conhecer a subcultura *geek*, como proposto neste ensaio teórico-reflexivo, favorece uma aproximação à TDUCC e pode ser um passo inicial no desenvolvimento da competência cultural, de modo que esta reflita claramente sobre a competência ética para uma abordagem mais humanística às pessoas estigmatizadas^(28)^. A conduta profissional na enfermagem, assim como em outras profissões, é uma expressão dos valores éticos que sustentam a profissão do(a) enfermeiro(a), dado que as suas atitudes e escolhas impactam diretamente a vida e o bem-estar das pessoas, além de influenciar a qualidade da assistência, o ambiente de trabalho e a relação de confiança entre a equipe e aqueles sob os seus cuidados. Nesse sentido, a competência ética desses profissionais manifesta-se na habilidade de analisar situações e agir conforme os princípios que asseguram a proteção, o respeito aos direitos, à dignidade e à integridade das pessoas^(30)^.

Outro aspecto observado na literatura, referente às pessoas estigmatizadas, é que as relações entre a pessoa e a família e entre a pessoa e o profissional de saúde, podem ser de superproteção ou negligenciadas. Ambas as circunstâncias podem gerar insegurança/medo pela falta de confiança na provisão de apoio^(28)^. Pesquisas sobre o *fandom furry*, por exemplo, mostram que os “*furries*”, ao buscar aconselhamento para condições físicas ou psicológicas, tiveram os seus problemas ignorados por vários profissionais de saúde, os quais, aliás, focaram no fato de “ser *furry*” como o problema^(18)^. É nesse cenário que as competências cultural e ética do(a) enfermeiro(a) fazem a diferença, de modo que o profissional apresentará as habilidades para intervir e criar espaços de cuidado, nos quais predominem a sensibilidade, o acolhimento, a escuta qualificada e a abertura para o diálogo, em um processo respeitoso e empático de comunicação. Assim, os que se sentem estigmatizados e suas famílias poderão conversar, externar seus medos ou dúvidas e propor outros olhares para a sociedade e para si mesmos^(25,28,31)^.

O cuidado de enfermagem é essencialmente ético na medida em que as ações são pautadas na integridade e as decisões tomadas priorizam os aspectos humanitários e o bem-estar das pessoas assistidas, buscando o seu melhor interesse. Contudo, a prática profissional diária dos(as) enfermeiros(as) é permeada de situações conflitantes e dificuldades, especialmente sobrecarga de trabalho, escassez de recursos e falta de apoio institucional, as quais podem limitar a prática ética. Nessa direção, a educação permanente em serviço, conforme já mencionado, tem sido reconhecida como um recurso valioso para o aprimoramento profissional, tanto do ponto de vista cultural, quanto ético. Enfermeiros(as) e equipes de enfermagem que participam de formação continuada sentem-se mais seguros e capacitados para atuarem em contextos “problemáticos” ou culturalmente divergentes, o que impacta positivamente na satisfação das pessoas assistidas e na retenção de profissionais nas instituições de saúde^(30)^.

Para além dos conceitos teóricos, as considerações aqui pontuadas de maneira reflexiva tiveram o propósito de desconstruir os rótulos das novas identidades como sendo “atípicas”, “não convencionais” ou “não-humanas” e despertar a enfermagem e os demais profissionais de saúde para o aperfeiçoamento das competências cultural e ética, a fim de ressignificar o cuidado às pessoas estigmatizadas por suas atividades, interesses e formas de interação social. Também é relevante enfatizar que o estigma social tem implicações na saúde daqueles que se sentem estigmatizados, demandando dos profissionais, habilidades interpessoais ainda mais complexas para a integralidade do cuidado. Assim, o autoconhecimento e a disposição pessoal para a mudança constituem os passos iniciais para a promoção da saúde, bem como para uma relação de respeito e confiança entre quem cuida e quem é cuidado^(31)^.

Ao apropriar-se do cuidado holístico, é possível refletir e compreender que os conceitos de normalidade e de estigmatização não se referem às pessoas em si, mas às perspectivas construídas em contextos sociais específicos. Essas compreensões emergem em interações mistas, sendo moldadas pela percepção do não cumprimento de normas sociais, que influenciam a dinâmica das interações, incluindo aquelas com os profissionais de saúde, dentre eles, os(as) enfermeiros(as) e equipes de enfermagem. Portanto, para mudar a realidade do estigma, é imperioso focar não nas diferenças, mas naquilo que nos faz semelhantes: somos todos seres humanos^(31)^.

## LIMITAÇÕES E CONTRIBUIÇÕES DO ESTUDO

As limitações referem-se à escassez de estudos na temática, sobretudo quanto ao cuidado de enfermagem no contexto das identidades apresentadas, o que dificultou o seu aprofundamento. Além disso, o uso de literatura cinzenta foi necessário por conter alguns aspectos fundamentais ao tema proposto. Outra limitação é quanto ao método escolhido, que não permite classificar a força das evidências identificadas.

Por outro lado, sua principal contribuição reside na redução da lacuna de conhecimento acerca do tema, especialmente no campo da saúde, ao clarificar as peculiaridades subculturais que, se ignoradas ou minimizadas, podem resultar em falhas na comunicação terapêutica, resistência ao cuidado ou insatisfação com o mesmo, sensação de não pertencimento nos espaços de atenção à saúde e inequidades no acesso e na qualidade do cuidado. Ademais, mediante os avanços da transformação digital na saúde, este ensaio propõe-se a problematizar a preparação dos profissionais de enfermagem para interagir de forma eficaz com subculturas profundamente integradas aos ambientes virtuais e comunidades on-line. Esse novo contexto digital desafia os modelos tradicionais de assistência, exigindo dos profissionais não apenas competência técnica, mas também cultural e a abertura para novas formas de mediação do cuidado.

## CONSIDERAÇÕES FINAIS

O presente ensaio teórico-reflexivo discorreu sobre as novas identidades, suas expressões e motivações e apresenta alguns *insights* acerca do cuidado de enfermagem nessa conjuntura. Acredita-se que as discussões decorrentes da reflexão podem possibilitar aos profissionais de saúde, especialmente, aos(às) enfermeiros(as) e equipes de enfermagem, a desmistificação de subculturas/comunidades virtuais, no intuito de transpor possíveis barreiras que podem surgir nas relações de cuidado, devido à estigmatização. Outras contribuições deste ensaio referem-se ao fomento de debates durante o percurso de formação profissional em saúde no que diz respeito à concepção de crenças, estereótipos, preconceitos e os fatores que favorecem a sua desconstrução. Por fim, espera-se fortalecer as competências culturais e éticas da enfermagem para ressignificar o processo de cuidar das pessoas estigmatizadas.

## Data Availability

O conjunto de dados que dá suporte suporte a este artigo não está disponível publicamente.

## Footnotes

*
*Fanfic* é um termo que deriva do inglês “*fan fiction*”, ou “ficção de fã”. São histórias de ficção criadas por fãs, baseadas no enredo e em personagens de filmes, séries, livros ou *games* ^(10)^.

# Translation: La atención de enfermería desde la perspectiva de las identidades subculturales: un ensayo reflexivo

**DOI:** 10.1590/1980-220X-REEUSP-2025-0140es

**Authors:** Lilian Cristiane Gomes, Maria Lúcia do Carmo Cruz Robazzi, Sérgio Valverde Marques dos Santos, Fábio de Souza Terra, Mônica La Salette da Costa Godinho, Cristiane Aparecida Silveira, Ana Kelley de Rezende, João Vitor Andrade, Namie Okino Sawada

**Affiliations:** 1 Universidade Federal de Alfenas, Escola de Enfermagem, Alfenas, MG, Brasil.

**Keywords:** Identificación Social, Diversidad Cultural, Estereotipo, Atención de Enfermería, Competencia Cultural

## Abstract

Este estudio teórico-reflexivo investigó la diversidad identitaria y la atención de enfermería, basándose en materiales seleccionados en PubMed/MedLine y FurScience, y ofreciendo una reflexión crítica sobre el tema. Se desarrollaron dos categorías: “Nuevas identidades: sus expresiones e implicaciones sociales”, que aborda la construcción de identidades sociales y subculturales en grupos con intereses comunes y cómo estas identidades influyen en la sociedad, y “La atención de enfermería desde la perspectiva de las nuevas identidades”, que destaca la importancia del conocimiento sobre las subculturas y las identidades sociales en la atención, especialmente con adolescentes y jóvenes. Esta comprensión favorece la humanización, fortalece el vínculo terapéutico y minimiza las barreras en la asistencia de enfermería. Las nuevas identidades se consideran construcciones socioculturales dinámicas, cuya reflexión teórica destaca la importancia de desmitificar los estigmas y promover una atención humanizada. Además, enfatiza las competencias culturales y éticas de la enfermería en la re-significación de la atención a las personas estigmatizadas, fomentando debates en la formación profesional en salud.

## INTRODUCCIÓN

En un mundo globalizado, impregnado de innovaciones y avances tecnológicos cada vez más rápidos y significativos^(1)^, surgen nuevas formas de socialización y agregación social que, a su vez, producen sus propias culturas o subculturas^(2,3)^. El término “subcultura” se refiere a los valores, creencias, actitudes, identidad y estilos de vida de un grupo dentro de la sociedad^(4)^. Como ejemplo, tenemos las subculturas “nerd” y “geek”, cuyos seguidores son personas excepcionalmente involucradas o interesadas, respectivamente, en las tecnologías/ciencias exactas y la cultura pop/mundos inventados (cómics, anime y manga, cosplay, entre otros), siendo especialistas en estas áreas. La subcultura*geek*, en particular, ha ganado gran popularidad y, en ella, cada área de interés constituye un “*fandom*”, es decir, una comunidad de fans o simpatizantes^(5–8)^.

Antes de la era digital, aquellos que se identificaban como “*geeks*” parecían aislados, tanto en sus intereses como de sus pares. Esto se debía a que muchas actividades*geek*(como leer, escribir y jugar) se consideraban inherentemente “menos sociales”, ya que, en la mayoría de los casos, se realizaban en soledad. Incluso los “*geeks*” que vivían en grandes ciudades tenían dificultades para encontrar y conectarse con otras personas que participaban en los mismos*fandom*s/comunidades^(7)^. El apego y la realización de estas actividades se consideraban, en cierta medida, un comportamiento antisocial o un signo de indiferencia hacia las interacciones sociales, lo que daba lugar a estereotipos negativos^(5,7,8)^. Sin embargo, la llegada de Internet permitió a las personas conectarse libremente, sin restricciones de tiempo, distancia, género o edad. Al igual que en la vida real, aquellos con intereses comunes comenzaron a reunirse en espacios virtuales para socializar. En general, los adolescentes y los jóvenes se adaptaron más rápidamente al nuevo escenario, ayudados por su capacidad para asimilar la tecnología con un mínimo esfuerzo. Así, se abrió un abanico de posibilidades para que los jóvenes eligieran cómo definirse y presentarse al mundo^(7)^.

Al reflexionar sobre el hecho de que los intereses y gustos son cambiantes, resulta comprensible que actividades que antes estaban mancilladas por un exceso de estigma social puedan ahora ganar capital social en línea^(7)^. Este hecho es tan genuino que, desde la última década, las actividades^(5)^.

Es en este contexto subcultural donde surge ampliamente la adopción de nuevas identidades, denominadas “no convencionales”, “atípicas” o “no humanas”^(9)^. Y, aunque el fenómeno de la globalización y la profusión de las tecnologías digitales han fomentado un mayor movimiento de ideas, así como una mayor circulación de personas e información^(1)^, aún se sabe poco sobre este universo “neo-identitario”. Cabe destacar que el término “nuevas identidades” empleado aquí no se refiere a su cronología/novedad temporal, sino a la escasa discusión que suscita en la literatura científica en el ámbito de la salud, lo que contribuye a perpetuar los prejuicios, los estereotipos y la desinformación. Esta situación, además de plantear cuestiones relevantes, exige la atención de los profesionales de la salud, especialmente de la enfermería, como profesión dedicada al cuidado integral del ser humano y, a menudo, responsable de la acogida inicial en los servicios de salud. Teniendo en cuenta la especificidad de sus funciones, es imprescindible que estos profesionales estén atentos a estas singularidades culturales para reconocer, comprender y respetar las prácticas e identidades de las subculturas digitales y mediáticas, como la*geek*, con el fin de establecer vínculos terapéuticos basados en una perspectiva empática y comprensiva con quienes cuidan.

De este modo, este estudio pretende no solo aclarar las definiciones subyacentes a las nuevas identidades y sus implicaciones sociales, sino, principalmente, proporcionar un marco teórico-reflexivo que pueda ayudar a los enfermeros y equipos de enfermería en el enfoque y la prestación de cuidados a las personas, así como en el apoyo y la aclaración a las familias, con el fin de prevenir y/o minimizar el sesgo de estigmatización y las posibles formas de sufrimiento psicoemocional. Así, el objetivo del presente estudio es analizar las nuevas identidades, las razones que las sustentan y sus repercusiones en la atención de enfermería.

## MÉTODO

Se trata de un ensayo teórico-reflexivo, elaborado mediante una revisión de la literatura, de forma narrativa y a partir de las siguientes inquietudes: ¿cuáles son las nuevas identidades y qué las influye y/o motiva? ¿Qué reflexiones surgen sobre los cuidados de enfermería en este contexto?

Para permitir una mejor comprensión sobre la adquisición de los materiales/textos utilizados, cabe destacar que la investigación se llevó a cabo en las siguientes fuentes de información: PubMed/MedLine y el sitio web*FurScience*. Se utilizaron como palabras clave/descriptores los términos: subcultura,*fandom*, identidad social, antropomorfismo y estigma, en los idiomas portugués e inglés, con el fin de buscar materiales más amplios que permitieran una mejor reflexión sobre este tema. La elaboración de este artículo se llevó a cabo tras la selección de los materiales bibliográficos relacionados con la temática mencionada, su lectura minuciosa con el fin de identificar los contenidos que se correspondían con los atributos investigados y la reflexión teórica sobre el tema, que sirvieron de base para la elaboración del estudio. Con ello, los principales puntos de discusión surgieron del análisis reflexivo basado en la literatura nacional e internacional, organizados en dos categorías: “Nuevas identidades: sus expresiones e implicaciones sociales” y “El cuidado de enfermería desde la perspectiva de las nuevas identidades”.

## DESARROLLO

### Nuevas Identidades: Sus Expresiones e Implicaciones Sociales

La identidad se entiende como un producto de la socialización a partir del contacto con el mundo y la transformación de la naturaleza, legitimada en la relación dialógica de la tríada persona/especie/sociedad. En las interacciones sociales, las personas desarrollan una identificación con los grupos a los que pertenecen mediante la internalización de normas, valores y rasgos culturales compartidos, proceso que contribuye a la formación del autoconcepto y orienta las conductas individuales. Estos grupos, a su vez, delinean sus propias subculturas, establecen las referencias constitutivas de su identidad colectiva y promueven el sentido de pertenencia entre sus miembros^(3,10)^. En este sentido, la identidad individual se yuxtapone a la social/cultural, concebida como el conjunto formado por el autoconcepto del individuo, su pertenencia grupal y la valoración atribuida a esta pertenencia^(2)^. Así, la identidad humana se presenta polimórfica en la medida en que cada persona es única y, al mismo tiempo, diversa en sus múltiples formas de expresión, dentro de la pluralidad cultural^(2,10)^.

Para algunos filósofos sociales, la subcultura^(13)^.

Existe un consenso sobre la importancia de la pertenencia para la construcción de la identidad y el desarrollo psicológico de los adolescentes y jóvenes. Los autores defienden que este sentido de pertenencia puede obtenerse y reforzarse mediante la participación en los fandoms geek. A través de la creación de vídeos y la participación en debates en línea, los jóvenes pueden encontrar un público receptivo a sus escritos o su música y adquirir nuevas habilidades para crear y construir. Pueden demostrar sus habilidades artísticas en forma de fotografías, artesanía o dibujos^(5,7)^. Por lo tanto, los*fandoms geek* proporcionan a los jóvenes la interacción en grupos con los que se identifican y desarrollan un sentido de orgullo por sus contribuciones a estas comunidades en crecimiento^(5,7,10)^.

Aunque el sentido de identidad y pertenencia puede reafirmarse al integrarse en fandoms/comunidades de la subcultura geek, dos de ellos destacan por tener un marcado estigma social, incluso entre los propios “geeks”, como son los *fandoms brony y furry* ^(7)^, que, aunque comparten un interés por los personajes/animales antropomórficos, son distintos entre sí^(14)^. Es debido a este interés que surgen, en los medios populares y de forma distorsionada, las etiquetas de “identidades atípicas”, “no convencionales” y “no humanas”.

El típico *brony* es un adolescente o joven adulto que forma parte del fandom de la serie de dibujos animados para niños My Little Pony: Friendship Is Magic. Los bronies describen su admiración por la serie como una apreciación del diseño estético y de los mensajes positivos de amistad y aceptación. Algunos críticos sugieren que esto representa una forma de “neo-sinceridad”: a pesar de que realmente les gustan estos elementos de la serie, los “bronies” presentan un sentido de ironía posmoderna y cinismo. Entienden que la sociedad convencional ve su admiración por dicha serie como algo desviado y, por lo tanto, utilizan la ironía como una forma de protección contra posibles reacciones negativas. El término “brony” es una combinación de “*bro*” (hipermasculinización de “*brother*”) y “*pony*”, símbolo típicamente femenino. La fusión entre la masculinidad y la identidad femenina simboliza la postura irónica del fandom. Las *fanfics* ^*^ basadas en los ponis son comunes, incluyendo el *clop-fic*, que explora narrativas eróticas. Muchos fans también producen arte inspirado en los ponis, algunos con tonos sexualizados. Este contenido puede contribuir en gran medida a la visión negativa que gran parte de la comunidad geek y de la sociedad en general tiene de los “bronies”^(7)^.

El ^(15)^.

La interacción entre los “furries” se produce tanto en el entorno virtual, a través de salas de chat, foros de discusión y juegos en línea, en los que avatares antropomórficos representan a los jugadores, como en persona, a través de convenciones. Hay eventos de esta naturaleza en las grandes ciudades, específicos para los “furries”, pero también participan en convenciones geek multigénero. El uso de un “^(19)^.

Los “*geeks*” de otros*fandom*s, que hacen una mayor distinción entre sus intereses y su identidad, consideran a los “*furries*” como extremos. En muchas comunidades, la marginación de los miembros más radicales se manifiesta como una estrategia para evitar que el grupo sea etiquetado o juzgado por observadores externos, basándose en los comportamientos y creencias de esta minoría. Un subgrupo dentro de la comunidad “*furry*”, conocido como “*yiffies*” o “*plushies*”, supuestamente participa en actividades sexuales mientras usa “f*ursuits*”. Aunque representan una minoría, el estigma asociado a ellos a menudo se generaliza a toda la comunidad “*furry*”^(7)^. En los medios de comunicación populares, este prejuicio se refuerza al retratar a los “furries” como un fandom fetichista o como un grupo de personas involucradas en comportamientos sexualmente desviados^(15,20)^.

Investigaciones recientes han demostrado que las motivaciones no sexuales son las más relevantes para la identidad social “*furry”*, principalmente el sentido de pertenencia y que, por lo tanto, los “*furries*” pueden teorizarse adecuadamente como un fandom centrado en los medios de comunicación con elementos sexuales, más que como una subcultura sexual con elementos de fandom^(15,16,20)^.

Muchos de los estereotipos negativos sobre el^(20)^.

Aunque existan motivaciones e intereses sexuales inusuales, este hecho no justifica la discriminación y la estigmatización^(16)^. Sin embargo, debido a esta posibilidad, los “^(7)^.

Otras comunidades emergentes son los “^(21)^.

Debido a las diferentes visiones que los “*furries*”, “*otherkins*” y “*therians*” tienen de sí mismos y entre ellos, aunque haya cierto solapamiento, los “*furries*” constituyen, en esencia, un*fandom*(comunidad de fans), mientras que los “*otherkins/therians*” son comunidades basadas en creencias y en la autoexpresión. Esto sugiere que la principal diferencia entre los “furries” y los otros dos grupos no radica necesariamente en el aprecio por los animales, sino en el grado en que sienten una conexión espiritual o identitaria más profunda con ellos^(22)^. Sin embargo, los investigadores consideran a los “*bronies*”, “*otherkins*”, “*therians*”, entre otros, como subgrupos del*fandom furry*. En sus estudios, estos investigadores han encontrado que el propio*fandom*señala la existencia de “*furries*problemáticos” y subgrupos controvertidos (como los mencionados anteriormente) y que esta percepción genera prejuicios, discriminación, intolerancia y acoso (*bullying)*también dentro del*fandom*, además del reconocimiento de que el acoso realmente existe. Aunque no sea físico, existe un*acoso*social significativo (por ejemplo, difundir rumores, practicar el ostracismo, entre otros), y la mayoría de los “*furries*” pasan por este tipo de experiencia en algún momento de su vida. Los casos de*acoso escolar*se produjeron con mayor frecuencia fuera del*fandom*y fueron más pronunciados entre los 11 y los 18 años, un periodo crítico para la formación de la identidad de una persona^(18,23)^.

Existe una vía de doble sentido que sustenta el círculo vicioso de los estereotipos negativos y el estigma social: por un lado, por temor al ridículo y otras repercusiones negativas, los “^(23)^.

En este contexto, el^(23)^.

Para los investigadores del IARP, la gente en general tiende a pensar que hay “algo malo” en los “furries”, una percepción que se refleja en las representaciones de los medios de comunicación y en los estereotipos negativos sobre este fandom, insistiendo en que sus miembros necesitan ser “explicados” en términos clínicos. Algunas personas sugieren la presencia de problemas psicológicos o de desarrollo. Otros se arriesgan a dar explicaciones situacionales, como una infancia difícil, tumultuosa, sin amigos o marcada por dificultades sociales. Después de todo, hay pruebas consistentes de que el acoso, el estigma y la ocultación de identidades estigmatizadas pueden ser especialmente perjudiciales para el bienestar de una persona. Por lo tanto, sería razonable esperar que los “furries” mostraran signos de bienestar significativamente comprometido. Sin embargo, los datos de la literatura sugieren lo contrario: no hay diferencias estadísticamente significativas en cuanto al bienestar, la autoestima y la satisfacción con la vida en comparación con la población general^(15,18)^, además del hecho de que los “*furries*” han demostrado un sentido de identidad más estable y coherente que los no*furries* ^(23,24)^.

Aunque tienen vidas fantásticas más activas, no son más propensos que otras personas a involucrarse en formas patológicas de fantasía (por ejemplo, ilusión, escapismo, fantasía excesiva); la tendencia a involucrarse es por razones saludables y beneficiosas, que incluyen la inspiración a la creatividad, la interacción social y la recreación. Los investigadores también destacan que, como resultado de la participación en una comunidad que valora la tolerancia, la aceptación y la mentalidad abierta, la interacción con el *fandom furry* se asocia con la ciudadanía global y un sentido de responsabilidad de actuar para mejorar el mundo (por ejemplo, preocupaciones medioambientales y valoración de la diversidad)^(15,18)^.

### La Atención de Enfermería Desde la Perspectiva de Las Nuevas Identidades

Al reflexionar sobre el hecho de que el proceso de atención en enfermería se centra principalmente en el ser humano, es innegable que la conducta profesional de la enfermería se basa en “la humanización y la acogida, sin distinción de personas”. Una mayor comprensión de la esencia cultural de las personas aclarará la percepción de quién es el paciente/cliente/usuario, minimizando las posibles barreras en la relación de cuidado, en particular aquellas que pueden surgir de los estereotipos. Conocer el estilo de vida, las costumbres y todo lo que rodea a las personas en sus subculturas constituye el primer paso para crear un vínculo terapéutico eficaz y prestar una atención humanizada y de calidad^(25)^.

Los enfermeros(as) y demás profesionales de la enfermería que atienden especialmente a adolescentes y jóvenes requieren una comprensión práctica de estas subculturas/comunidades, en su mayoría ocultas. El interés de un joven por participar en un^(7)^.

En las culturas occidentales, es muy común que los aficionados al deporte vistan la camiseta de su equipo en un evento especial, como un partido, o que un fanático de la música lleve la camiseta de su banda favorita. Lo mismo ocurre con los miembros de los^(23)^.

En este contexto, la atención de enfermería debe orientarse, en primer lugar, a romper con la tendencia a la “patologización”. A pesar de la suposición implícita en los estereotipos de que solo las personas con sufrimiento emocional o propensas a “problemas psíquicos” se involucran en actividades “geek” o que tal implicación da lugar a esas condiciones, no hay pruebas que respalden esta suposición^(5,8,20,23)^. Por el contrario, los estudios indican que los “*geeks*” muestran un mayor compromiso cívico y no presentan déficits en cuanto a pertenencia, tamaño de la red social u orientación hacia el futuro. Así, existe un consenso de que son diferentes, pero no disfuncionales^(5,7–8,23)^, por lo que requieren una mayor atención por parte de los profesionales de la salud, especialmente de enfermería, profesión que está directamente relacionada con la población^(25)^. Esta comprensión permite deshacerse de los prejuicios y los estereotipos negativos que rodean a los*fandom geeks*.

Al ser una subcultura que comparte sus intereses casi siempre a través de foros en línea*,*redes y plataformas de redes sociales, el uso de tecnologías digitales puede facilitar el acercamiento a esta clientela específica. Además, en entornos virtuales, los enfrentamientos que dan lugar al*acoso/ciberacoso*son más frecuentes, ya que las plataformas de redes sociales no pueden supervisar todos los mensajes publicados por sus usuarios. El supuesto anonimato de las redes sociales hace que algunos usuarios difundan el odio o eviten comportamientos civilizados, con la falsa percepción de que no existen consecuencias reales para estos actos^(7)^. La incorporación de estas tecnologías como canales de escucha activa y cualificada se considera una estrategia interesante para la atención de enfermería, ya que permite a los profesionales identificar rápidamente a las personas que necesitan apoyo, así como las posibles intervenciones y derivaciones.

Otro aspecto a considerar en la atención se refiere a la inclusión del ocio para establecer relaciones más cercanas y acogedoras con las personas, respetando su subjetividad e identidad cultural. Las actividades de ocio se proponen satisfacer las necesidades personales y producen efectos distintos según los valores y objetivos de cada persona. Lo esencial en esta práctica es contribuir al desarrollo de las potencialidades individuales y promover el bienestar^(26)^. Las actividades*geek*cumplen este papel al trascender el mero entretenimiento, ya que para sus adeptos representan mecanismos significativos de socialización y construcción de la identidad. También impulsan la creatividad y actúan como fuentes relevantes de resiliencia emocional^(24)^, contribuyendo así a la consolidación de la confianza interpersonal^(15)^. Por lo tanto, corresponde a los profesionales de enfermería estimular las actividades de ocio que promueven la autonomía y la satisfacción personal. Este enfoque está en consonancia con la literatura sobre estrategias de afrontamiento a través del ocio (*leisure coping*)^(5)^.

La promoción de entornos clínicos acogedores y simbólicamente inclusivos es otra herramienta de acercamiento cultural, imprescindible para romper el estigma en la comunidad y dentro de los servicios de salud. Esto puede incluir desde la flexibilización del lenguaje utilizado en la atención hasta la exhibición de señales visuales que demuestren familiaridad y respeto por la subcultura*geek*, como carteles con personajes populares y el uso de tecnologías interactivas o de gamificación. Este entorno puede reforzar el vínculo terapéutico, favorecer la comunicación y minimizar las barreras culturales que dificultan la relación profesional-paciente^(27)^. En cuanto a este último aspecto, mostrar curiosidad genuina por los intereses de la persona, utilizando los elementos de su subcultura como facilitadores en la construcción de esta relación, así como comprender que puede utilizar referencias de juegos, películas o personajes como mecanismos de afrontamiento emocional, contribuye a una conducta profesional más empática e individualizada.

También es responsabilidad de enfermería asumir el papel educador, proporcionando información basada en la evidencia sobre las subculturas, los *fandoms* y sus respectivas identidades, actividades e intereses. Fomentar tertulias, charlas, campañas de sensibilización y apoyar iniciativas que promuevan la equidad y el respeto a la diversidad son estrategias indispensables para reducir los prejuicios sociales^(28)^. Para ello, es necesario que estos profesionales de la salud adquieran y/o mejoren su competencia cultural, ya que es fundamental que dicha competencia se incorpore de manera más profunda y sistemática en su formación académica, a fin de prepararlos para el mercado laboral con una comprensión más amplia de la diversidad cultural. De esta manera, se evita que el contacto con realidades diferentes genere incomodidad hasta el punto de comprometer la práctica profesional. Además, es esencial promover la educación permanente en el servicio a lo largo de la trayectoria laboral, con el fin de mejorar las habilidades de interacción con diferentes contextos culturales. Esta inversión continua permite que, incluso ante retos y barreras, los enfermeros, técnicos y auxiliares de enfermería sean capaces de desarrollar soluciones eficaces y sensibles a las especificidades culturales/subculturales^(25)^.

Un punto de partida para desarrollar plenamente la competencia cultural es apropiarse de los supuestos de la Enfermería Transcultural a través de la Teoría de la Diversidad y Universalidad del Cuidado Cultural (TDUCC), de Madeleine Leininger. Esta teoría tiene como objetivo proporcionar una forma de cuidado que sea sensible y se base en las necesidades de la persona, su familia y los grupos culturales a los que pertenece. Para ello, es esencial conocer las peculiaridades de estos grupos, con el fin de identificar similitudes y diferencias en relación con los cuidados y los comportamientos de salud. Este conocimiento permite desarrollar una atención culturalmente adecuada y coherente con las necesidades específicas de una población determinada. Los enfermeros, técnicos y auxiliares de enfermería dotados de competencia cultural perfeccionan con eficacia su capacidad de comunicación, establecen relaciones horizontales con quienes cuidan en la acción, en la escucha y en el pensamiento, y despiertan el compromiso de las personas en los cuidados en salud al hacerles creer que son valoradas y respetadas en su forma de vivir^(29)^.

Conocer la subcultura^(30)^.

Otro aspecto observado en la literatura, en relación con las personas estigmatizadas, es que las relaciones entre la persona y la familia y entre la persona y el profesional de la salud pueden ser de sobreprotección o de negligencia. Ambas circunstancias pueden generar inseguridad/miedo por la falta de confianza en la prestación de apoyo^(28)^. Las investigaciones sobre el*fandom* *furry*, por ejemplo, muestran que los “*furries*”, al buscar asesoramiento para condiciones físicas o psicológicas, vieron ignorados sus problemas por varios profesionales de la salud, quienes, además, se centraron en el hecho de “ser*furry*” como el problema^(18)^. Es en este contexto donde las competencias culturales y éticas del enfermero marcan la diferencia, de modo que el profesional tendrá las habilidades para intervenir y crear espacios de atención en los que predominen la sensibilidad, la acogida, la escucha cualificada y la apertura al diálogo, en un proceso de comunicación respetuoso y empático. Así, quienes se sienten estigmatizados y sus familias podrán conversar, expresar sus miedos o dudas y proponer otras miradas hacia la sociedad y hacia sí mismos^(25,28,31)^.

El cuidado de enfermería es esencialmente ético en la medida en que las acciones se basan en la integridad y las decisiones tomadas priorizan los aspectos humanitarios y el bienestar de las personas atendidas, buscando su mejor interés. Sin embargo, la práctica profesional diaria de los enfermeros está plagada de situaciones conflictivas y dificultades, especialmente la sobrecarga de trabajo, la escasez de recursos y la falta de apoyo institucional, que pueden limitar la práctica ética. En este sentido, la educación permanente en el servicio, como ya se ha mencionado, ha sido reconocida como un recurso valioso para el perfeccionamiento profesional, tanto desde el punto de vista cultural como ético. Los enfermeros y los equipos de enfermería que participan en la formación continua se sienten más seguros y capacitados para actuar en contextos “problemáticos” o culturalmente divergentes, lo que repercute positivamente en la satisfacción de las personas atendidas y en la retención de los profesionales en las instituciones sanitarias^(30)^.

Más allá de los conceptos teóricos, las consideraciones aquí señaladas de manera reflexiva tenían el propósito de deconstruir las etiquetas de las nuevas identidades como “atípicas”, “no convencionales” o “no humanas”, y despertar a la enfermería y a otros profesionales de la salud para el perfeccionamiento de las competencias culturales y éticas, con el fin de redefinir la atención a las personas estigmatizadas por sus actividades, intereses y formas de interacción social. También es importante destacar que el estigma social tiene implicaciones en la salud de quienes se sienten estigmatizados, lo que exige a los profesionales habilidades interpersonales aún más complejas para la integralidad de la atención. Así, el autoconocimiento y la disposición personal al cambio constituyen los primeros pasos para la promoción de la salud, así como para una relación de respeto y confianza entre quienes cuidan y quienes son cuidados^(31)^.

Al apropiarse del cuidado holístico, es posible reflexionar y comprender que los conceptos de normalidad y estigmatización no se refieren a las personas en sí mismas, sino a las perspectivas construidas en contextos sociales específicos. Estas comprensiones surgen en interacciones mixtas y están moldeadas por la percepción del incumplimiento de las normas sociales, que influyen en la dinámica de las interacciones, incluidas las que se establecen con los profesionales de la salud, entre ellos los enfermeros(as) y equipo de enfermería. Por lo tanto, para cambiar la realidad del estigma, es imperativo centrarse no en las diferencias, sino en lo que nos hace similares: todos somos seres humanos^(31)^.

## LIMITACIONES Y CONTRIBUCIONES DEL ESTUDIO

Las limitaciones se refieren a la escasez de estudios sobre el tema, sobre todo en lo que se refiere a la atención de enfermería en el contexto de las identidades presentadas, lo que dificultó su profundización. Además, fue necesario recurrir a la literatura gris, ya que contiene algunos aspectos fundamentales para el tema propuesto. Otra limitación es el método elegido, que no permite clasificar la solidez de las pruebas identificadas.

Por otro lado, su principal contribución radica en la reducción de la brecha de conocimiento sobre el tema, especialmente en el campo de la salud, al aclarar las peculiaridades subculturales que, si se ignoran o minimizan, pueden dar lugar a fallos en la comunicación terapéutica, resistencia a los cuidados o insatisfacción con los mismos, sensación de no pertenencia a los espacios de atención sanitaria y desigualdades en el acceso y la calidad de los cuidados. Además, mediante los avances de la transformación digital en la salud, este ensayo se propone problematizar la preparación de los profesionales de enfermería para interactuar de manera eficaz con subculturas profundamente integradas en los entornos virtuales y las comunidades en línea. Este nuevo contexto digital desafía los modelos tradicionales de asistencia, exigiendo a los profesionales no solo competencia técnica, sino también cultural y apertura a nuevas formas de mediación en el cuidado.

## CONSIDERACIONES FINALES

El presente ensayo teórico-reflexivo ha abordado las nuevas identidades, sus expresiones y motivaciones, y presenta algunas*ideas*sobre la atención de enfermería en este contexto. Se cree que las discusiones derivadas de la reflexión pueden permitir a los profesionales de la salud, especialmente a los enfermeros y equipos de enfermería, desmitificar las subculturas/comunidades virtuales, con el fin de superar las posibles barreras que pueden surgir en las relaciones de cuidado debido a la estigmatización. Otras contribuciones de este ensayo se refieren al fomento de debates durante la formación profesional en salud en lo que respecta a la concepción de creencias, estereotipos, prejuicios y los factores que favorecen su deconstrucción. Por último, se espera fortalecer las competencias culturales y éticas de la enfermería para resignificar el proceso de cuidado de las personas estigmatizadas.

## Data Availability

El conjunto de datos que respalda este artículo no está disponible públicamente.

## Footnotes

*
*Fanfic* es un término que deriva del inglés “*fan fiction*”, o “ficción de fans”. Son historias de ficción creadas por fans, basadas en el argumento y los personajes de películas, series, libros o *videojuegos* ^(10)^.
